# The Person-centred Nursing Framework: a mid-range theory for nursing practice

**DOI:** 10.1177/17449871241281428

**Published:** 2025-03-13

**Authors:** Tanya McCance, Brendan McCormack

**Affiliations:** Mona Grey Professor of Nursing R&D, School of Nursing and Paramedic Sciences, Ulster University, Belfast, UK; The Susan Wakil Professor of Nursing, Head of School & Dean, The Susan Wakil School of Nursing and Midwifery (inc. Sydney Nursing School), Faculty of Medicine and Health, The University of Sydney, Camperdown, NSW, Australia

**Keywords:** nursing education, nursing practice, patient experience, practice development, person-centredness, research impact, theory

## Abstract

**Background:**

Person-centredness is a global movement in healthcare that prioritises the human experience. Person-centred care has a long association with nursing; however, the implementation of person-centredness as a philosophy of practice remains challenging and requires a sustained focus on the development of healthful workplace cultures.

**Aim:**

This is a discussion paper that focuses on the theoretical development of person-centred nursing, drawing out the uniqueness of the Person-centred Nursing Framework (PCNF) to the discipline of nursing and its relevance as a middle-range theory for nursing practice.

**Discussion:**

The PCNF highlights the complexity of person-centred nursing, and through the articulation of the key constructs, emphasises the contextual, attitudinal and moral dimensions of humanistic caring practices. The development of the framework as a means of operationalising person-centredness in practice has been integral to the establishment of a research programme spanning over two decades. The programme has privileged research that focuses on the implementation of person-centred nursing in a variety of healthcare contexts, illustrating outcomes that focus on enhanced care experience for both patients and nurses.

**Conclusions:**

The theoretical development of nursing through the lens of the PCNF highlights the uniqueness of person-centredness to the discipline of nursing.

## Introduction

Person-centredness is a global movement in healthcare because it reflects the importance of keeping the person at the centre of healthcare systems. It prioritises the human experience and places compassion, dignity and humanistic caring principles at the centre of healthcare delivery that are translated through relationships that are built on effective interpersonal processes (McCormack, 2020). It reflects the philosophical discourse of moving away from biomedical approaches to healthcare, towards those broadly termed as psycho-social and views humans as persons situated within their own context (Phelan et al., 2020).

Person-centred care is a term that is being used to describe a standard of care that ensures the patient is at the centre of care delivery. Person-centred care has a long association with nursing, and at a level of principle is well understood as that which is concerned with: treating people as individuals; respecting their rights as a person; building mutual trust and understanding; developing therapeutic relationships (McCormack and McCance, 2006). As nurses we have an expectation that people should receive a standard of care that reflects these principles. The inherent good of providing care within a philosophy of person-centredness is irrefutable, but it has been recognised that translating the core concepts into every day practice is challenging. The reasons for this come in many forms and are often indicative of the context in which care is being delivered, and the fact that we are living in times of constant change, particularly within health and social care (Baldie et al., 2021).

The body of evidence relating to person-centredness in healthcare is significant. The focus of attention in the current literature is on the development of person-centredness in practice (as opposed to solely focusing on person-centred care), and the strategies required to overcome the cultural and contextual challenges to implementing a person-centred approach (McCormack et al., 2021). Significantly, this agenda is being further fuelled by the increasing global challenges across our healthcare systems, particularly post-pandemic (Maben et al., 2022). For nursing the headline is unprecedented workforce shortages, further impacted by reports of low job satisfaction, low morale and burnout, reflecting workplace cultures that are not healthful (NHS Resolution, 2023). Furthermore, we know from the evidence, however, that systematically developing person-centred practice leads to increased team effectiveness, increased staff morale, enhanced job satisfaction and better retention of staff (Haraldsdottir et al., 2020; Lynch and McCance, 2022; McCormack et al., 2018). It is therefore not surprising that in the current context, senior nursing and midwifery leaders are re-evaluating their approach to developing person-centred cultures and placing it as the central theoretical underpinning for strategy development. Whilst there are many examples of person-centred principles and practices being embedded in health systems globally (cf. The WHO website https://www.who.int/health-topics/integrated-people-centered-care#tab=tab_1), two particular examples from Australian and United Kingdom contexts illustrate the importance of embedding implementation strategies for person-centredness in the macro context, thus taking account of factors that are political and strategic in nature (SESLHDN&MStrategyPlan2023-26 Final.pdf (nsw.gov.au); Five-year vision outlined for Nursing & Midwifery, Department of Health (health-ni.gov.uk). Whilst these examples highlight the commonalities that exist in the macro policy context at a global level, they also highlight the importance and significance of the need for systematic translation of these macro agendas into practical action in specific organisations and systems.

Whilst there is some evidence of impact as highlighted in this paper, there is also increasing awareness of the challenges that make it difficult to systematically achieve person-centredness across healthcare environments. These include: the problem of language; competing concepts and theories; policy and strategy weaknesses; weak macro and mezzo organisational frameworks (McCormack, 2024). Interestingly, there is a school of thought emerging that suggests ‘what counts as being person-centred can vary across different care contexts’ (Mitchell et al., 2022: 1).

In this discussion paper, the Person-centred Nursing Framework (PCNF) developed by McCormack and McCance (2006, 2010, 2021) will be presented. It focuses on the theoretical development of person-centred nursing over time, drawing out the uniqueness of the PCNF and its relevance as a middle-range theory for nursing practice. This will be placed in the context of the origins of the framework, which are founded on the concepts of caring and person-centredness. The theory is viewed as a means of operationalising person-centred nursing, which is neither impacted by a specific context, nor the amount of time spent caring for patients and their families. The essence of person-centredness is described as a way of being and focuses on ways of engaging that are necessary to create connections between persons in any one caring moment. Furthermore, throughout the evolution of the PCNF, it has been used in a variety of ways including: as a tool for reflection, as the theoretical positioning in research, to guide implementation studies, as a foundation for the development of instruments to measure person-centred practice, as a framework that underpins healthcare curricula and to underpin strategy and policy frameworks. In this paper, the position of the PCNF as a middle-range theory will be explored and placed in the context of the metaparadigm of nursing, thus drawing out its uniqueness to the discipline of nursing.

## The evolution of the PCNF

As a preface to presenting the components of the PCNF, it is important to highlight its evolution and place this in the context of theory development for the discipline of nursing. Fawcett (1995) described a hierarchy of nursing knowledge that has five components: the metaparadigm, philosophies, conceptual models, theories (grand and mid-range) and empirical indicators. We have used Fawcett’s (1995) seminal work on the hierarchy of nursing knowledge to chart the development of the PCNF as a mid-range theory for nursing practice.

The PCNF was derived from two original doctoral studies. McCormack’s study aimed to explore the meaning of autonomy for older people in hospital settings. His research combined the hermeneutic philosophy of Gadamer (1993) with conversational analysis to gain a deep understanding of how registered nurses facilitated autonomy in decision-making with older people. He focused on discharge planning as a particular focus of decision-making. It was through the analysis of these naturally occurring conversations that the philosophy of ‘personhood’ was identified as an essential factor in autonomous decision-making. The research resulted in the development of a conceptual framework for person-centred practice with older people called the ‘Authentic Consciousness Framework’ that elevated respect for personhood as a central component of nursing practice (McCormack, 2001, 2003). McCance’s study aimed to explore patients’ experience of caring provided by qualified nurses during an inpatient stay in medical and surgical units in a large acute general hospital. The rationale underpinning this study reflected the centrality of caring to nursing practice and a desire to elucidate how key constructs in the process of caring interact to produce a positive patient outcome. At the time of this study, there was a growing body of literature focusing on the concept of caring and theoretical developments of relevance to nursing (McCance et al., 1999). The key outcome from this study was the development of a conceptual framework for caring in nursing practice (McCance, 2003). This conceptual framework offered an original perspective on caring in nursing practice, which linked caring and quality of care, defined through the patient experience. At this stage, the focus was on two separate conceptual frameworks, which according to Fawcett (1995), provide a particular frame of reference that says something about ‘how to observe and interpret the phenomena of interest to the discipline’ (p. 3).

McCormack and McCance then came together to work on a large quasi-experimental study that focused on measuring the effectiveness of the implementation of person-centred nursing in a tertiary hospital setting (McCormack et al., 2008). It was during the intervention stage of this study that the PCNF was developed as a mechanism to shape and evaluate the person-centred nursing developments across the clinical settings. McCormack and McCance recognised the complementary foci of the frameworks through the following perspectives:

- They were derived from a humanistic perspective of caring.
- Initial review of the frameworks indicated a high degree of consistency across individual concepts and thus a high degree of face validity.
- They were both derived from inductive and systematic collaborative research processes.
- Collectively, they represented a synthesis of the then-available literature on caring and person-centredness (McCormack and McCance, 2010).

However, in addition to the synergy between the two conceptual frameworks, there was strongly shared philosophical underpinnings that were rooted in human science and what it means to be a person. The principles underpinning these two conceptual frameworks were consistent with human science principles such as those articulated by Watson (1985), including the centrality of human freedom, choice and responsibility; holism (nonreducible persons interconnected with others and nature); different forms of knowing (empirics, aesthetics, ethics and intuition); the importance of time and space, and relationships (McCormack and McCance, 2006). It was at this stage that the PCNF met the criteria as a middle-range theory for nursing as described by Fawcett (1995). Theories in Fawcett’s hierarchy of nursing knowledge are less abstract and can be further described as grand theories or middle-range theories, with the latter being narrower in scope and ‘made up of concepts and propositions that are empirically measurable’ (Fawcett, 1995: 25). Fawcett distinguishes between conceptual models and mid-range theories; in that, mid-range theories articulate one or more relatively concrete and specific concepts that are derived from a conceptual model, with propositions that describe these concepts and propose specific relationships between them.

The period following the publication of the original framework was characterised by wide exposure to the framework mainly within nursing, but on an international stage. The focus was to generate much needed critical dialogue and debate about its applicability to practice. The key message at this time was the utility of the framework as a means of operationalising person-centredness in nursing practice. The framework became increasingly recognised as a tool that shone a light on practice and brought a shared understanding and a common language to person-centredness in nursing. The publication of the PCNF in the first edition of the book (McCormack and McCance, 2010) consolidated many of the constructs within the framework, and the relationships between them. Following this publication, the framework continued to be used as a tool for practice and tested through ongoing research (e.g. Buckley et al., 2014; Lynch et al., 2011; McCance et al., 2013). At this stage, the PCNF became a recognised model for nursing and was included as the United Kingdom contribution in a text focusing on global perspectives for conceptual models of nursing (McCormack and McCance, in Fitzpatrick and Whall, 2016). It was in this publication that the PCNF was explicitly aligned to the four concepts within the metaparadigm of nursing (person, nursing, environment and health), situating it firmly within the hierarchy of nursing knowledge.

The Framework continued to evolve to take account of a wider engagement from other stakeholders, which resulted in the publication of the Person-centred Practice Framework (PCPF) (2017). The PCPF was placed within a broader context to illustrate its applicability to a wide range of healthcare workers. This was undoubtedly a positive development and supported much needed conversations regarding the development of person-centred practice at systems level. The framework largely remained stable over time, with subtle changes made to some components between each iteration, which reflected critical dialogue with a wider range of healthcare professionals. At this point, the PCPF (McCormack and McCance, 2017) was the version being most widely used, despite developments in education, practice and research that were specific to nursing. More recently, a further refresh of the PCPF was undertaken to firmly place it within a multi-professional context (McCance and McCormack, 2021). At the same time, it was decided to also revise and refresh the PCNF, which not only reflected it’s nursing roots, but retained and privileged it as an accepted conceptual model for nursing (McCormack and McCance, 2021). The uniqueness of the PCNF and PCPF is well rehearsed in the published literature. To summarise, it is a theoretical model that offers a vehicle to operationalise person-centredness in practice and has a strong philosophical basis rooted in humanistic science with the concept of personhood at its core. Furthermore, its focus is broader than engaging in person-centred care with patients and families, with an explicit emphasis on person-centredness being a concept that applies equally to all persons and is best achieved in healthful workplace cultures that enable everyone to flourish and reach their full potential. With this background context in mind, the PCNF will now be presented using the metaparadigm of nursing.

## PCNF: A mid-range theory

The PCNF comprises five domains:

1. *Metaparadigm* focuses on the four dimensions of nursing knowledge – person, nursing, environment and health.
2. Prerequisites focus on the attributes of the nurse, represented by the constructs: being professionally competent, having developed interpersonal skills, being committed to the job, being able to demonstrate clarity of beliefs and values and knowing self.
3. The care environment focus on the complexity of the context in which nursing care is experienced, represented by the constructs: appropriate skill mix; systems that facilitate shared decision-making; power sharing; effective staff relationships; organisational systems that are supportive; the potential for innovation and risk taking and the physical environment.
4. Person-centred nursing processes focus on ways of engaging that are necessary to create connections between nurses, patients and others significant to them in their lives through the constructs of, working with persons’ beliefs and values; engaging authentically, being sympathetically present; sharing decision making and providing holistic nursing care.
5. Expected outcome is the results of effective person-centred nursing and is simply stated as ‘a good care experience’ (for patients, families and nurses).

**Figure 1.**
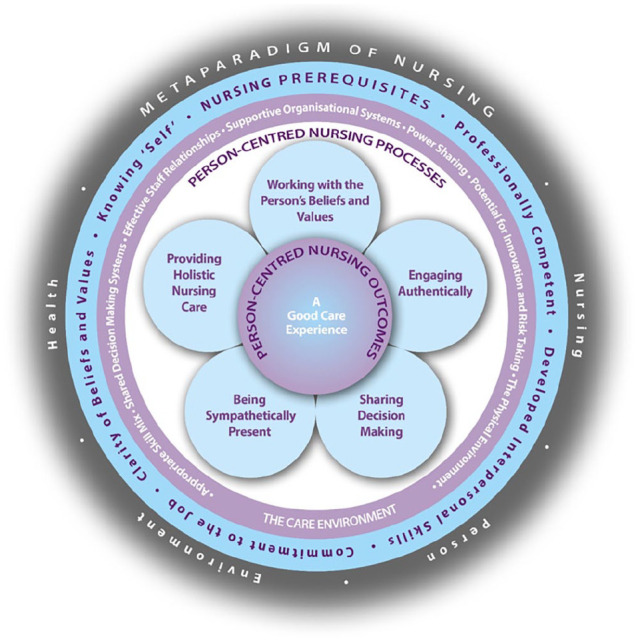
Person-centred Nursing Framework.

The relationship between the four domains of the framework is indicated by the pictorial representation, the idea being, you need to work from the outer circle inwards. In other words, the attributes of the nurse must first be considered as a prerequisite to managing the care environment, to provide effective care through the person-centred nursing processes and to achieve the outcome. It is also acknowledged that there are relationships between constructs within and across domains. Finally, the framework sits within the foundations of nursing knowledge (the fifth domain), reflecting the *metaparadigm of nursing*. The subsequent sections will discuss the components of the framework in relation to the four concepts of the metaparadigm. The most recent version of the framework is presented in Figure 1.

### Concept of person

The concept of person is central to the PCNF and captures those attributes that represent our humanness and the way in which we construct our life. How we think about moral values, how we express political, spiritual or religious beliefs, how we engage emotionally and, in our relationships, the kind of life we want to live, are all shaped by our attributes as persons.

We suggest that a person is *an authentic interacting being.* Whilst authenticity can be seen to be a unifying principle, we need to be acutely aware of the fact that many people are unable to represent their authentic self autonomously and so need help from others in situations where their authenticity may be under threat. Having some understanding of another’s beliefs, values, hopes, dreams and desires enables us to potentially provide such help to another person. Think about our closest relationships – in these relationships we share our deepest sense of ‘self’ with others, enabling others to know who I am as a person, what values are important to me, the dreams, hopes and desires I hold in my life and the kind of life that I strive to live. ‘Knowing the person’ in this way is essential to enacting personhood and knowing how to maximise a person’s autonomy is a key consideration in person-centred nursing. Having some sense of what authenticity means to a person and how that manifests itself through their being-in-the-world is essential to providing person-centred care. Through our discussions, reflections, debates, arguments and agreements our knowing of the person is shaped and reshaped, ordered and reordered, prioritised and reprioritised as life progresses. It is this authenticity of persons that should shape a plan of care that is genuinely authentic and person-centred.

Throughout life, persons continually grow, develop and experience transition and so there is always the potential for a new direction in life to be taken. So long as this new direction is consistent with the person’s authenticity, then the evolving-self can be accommodated. If a person’s life contains conflicting traits and goals, then they can be paralysed by confusion and ambivalence and may not be able to act with confidence that the action represents their authentic self. Reflection on the effectiveness of decisions is always considered against authenticity and the making of authentic choices. We contend that an authentic plan of care needs to help another person enact their different modes of being.

*Being in relation* emphasises the importance of relationships and the interpersonal processes that enable the development of relationships that have therapeutic benefit. *Being in a social world* considers persons to be interconnected with their social world, creating and recreating meaning through their being in the world. Closely linked to being in a social world is *being with self*, which emphasises the importance of persons ‘knowing self’ and the values they hold about their life and how they make sense of what is happening to them. *Being in place* encourages us to pay attention to ‘place’ recognising the impact of the ‘milieu of care’ on the care experience.

### Concept of nursing

The essence of nursing depicted within the framework reflects the ideals of humanistic caring, where there is a moral component and practice has at its basis a therapeutic intent, which is translated through relationships that are built upon effective interpersonal processes (McCormack and McCance, 2010). Therefore, person-centredness in nursing practice requires the formation of therapeutic relationships between professionals, patients and others significant to them in their lives, and these relationships are built on mutual trust, understanding and a sharing of collective knowledge (Dewing, 2004; McCormack, 2001; McCormack and McCance, 2010). The definition used in the framework was developed in a National Nursing Action Research Program in Ireland, which at that time closely reflected this literature and is consistent with the understandings of person-centredness in a nursing context:

. . .an approach to practice established through the formation and fostering of healthful relationships between all care providers, service users and others significant to them in their lives. It is underpinned by values of respect for persons, individual right to self-determination, mutual respect and understanding. It is enabled by cultures of empowerment that foster continuous approaches to practice development (McCormack and McCance, 2017: 3)

The framework highlights the complexity of person-centred nursing, and through the articulation of the key constructs, emphasises the contextual, attitudinal and moral dimensions of humanistic caring practices. The relationship between the constructs describes the necessity for competent nurses, who have the ability to manage the numerous contextual and attitudinal factors that exist within care environments, to engage in processes that keep the person at the centre of caring interactions (McCormack and McCance, 2021).

With the lens on developing competent nurses, significant attention has been given to developing person-centredness in the curriculum. Cook et al. (2018) undertook a longitudinal cohort study to explore the development of caring attributes in preregistration nursing students through this person-centred focused curriculum. This study tracked how University preregistration nursing students (*N* = 212) developed their caring attributes over their 3-year programme using the Caring Dimensions Inventory, a 35-item instrument, with 25 caring items under three constructs: technical, intimacy and supporting (Watson et al., 2001). Results showed that students developed their caring attributes throughout their programme, ranking 22 of 25 items as caring (with statistical significance) at the end of year 1, 18 at the end of year 2 and all 25 caring items at the end of their final year. This study found caring attributes can not only be sustained but can also be developed throughout a preregistration nursing education programme grounded in person-centredness. More recently, O’Donnell’s (2021) mixed methods study demonstrated how on completion of their studies, students’ perceptions of their person-centred practice were improved, having experienced a person-centred curriculum. However, it is important to acknowledge that both these studies are single site studies, situated in one higher education institute whose curriculum is underpinned by person-centred principles, with the PCNF as the golden thread that runs throughout the course structure and delivery. Additionally, the experience of engaging in such a curriculum may increase the students’ awareness of socially desirability in responses and may also be influenced by the power dynamic that can exist between researcher/lecturer and students.

Further development of person-centredness in curricula has been undertaken with an international collaborative through an Erasmus+ funded project. The collaborative comprised five higher education institutes across United Kingdom and Europe with experience of integrating person-centredness in curricula (Cook et al., 2022; Dickson et al., 2020; Phelan et al 2020). This has culminated in a Person-centred Curriculum Framework that can support teams to engage in critical discussions about curriculum content, the systems needed to enable meaningful learning and the cultures required to facilitate effective learning and development (McCormack 2024: 23–24). This framework points to areas for further research into person-centredness in curricula at systems level, which will be instrumental for developing person-centred healthcare practitioners.

The care processes also reflect the concept of nursing and the knowledge and skills required by nurses to create connections between nurses, patients and others significant to them in their lives. It could be argued, however, that there is no difference in how the person-centred processes are presented between the PCNF and its counterpart, the PCPF. This is not surprising given the fundamental basis of person-centred healthcare practices. However, as highlighted by Fitzpatrick and Whall (2016), the description of nursing as a metaparadigm concept focuses on actions taken in relation to the goals of nursing, set within the overall view of society, and articulates the ways in which nursing addresses the care of individuals, families, groups and communities. The PCNF operationalises the practice of nursing, as distinct from other disciplines, through the person-centred nursing processes, with an explicit focus on the ultimate goal – a good care experience.

**Figure 2.**
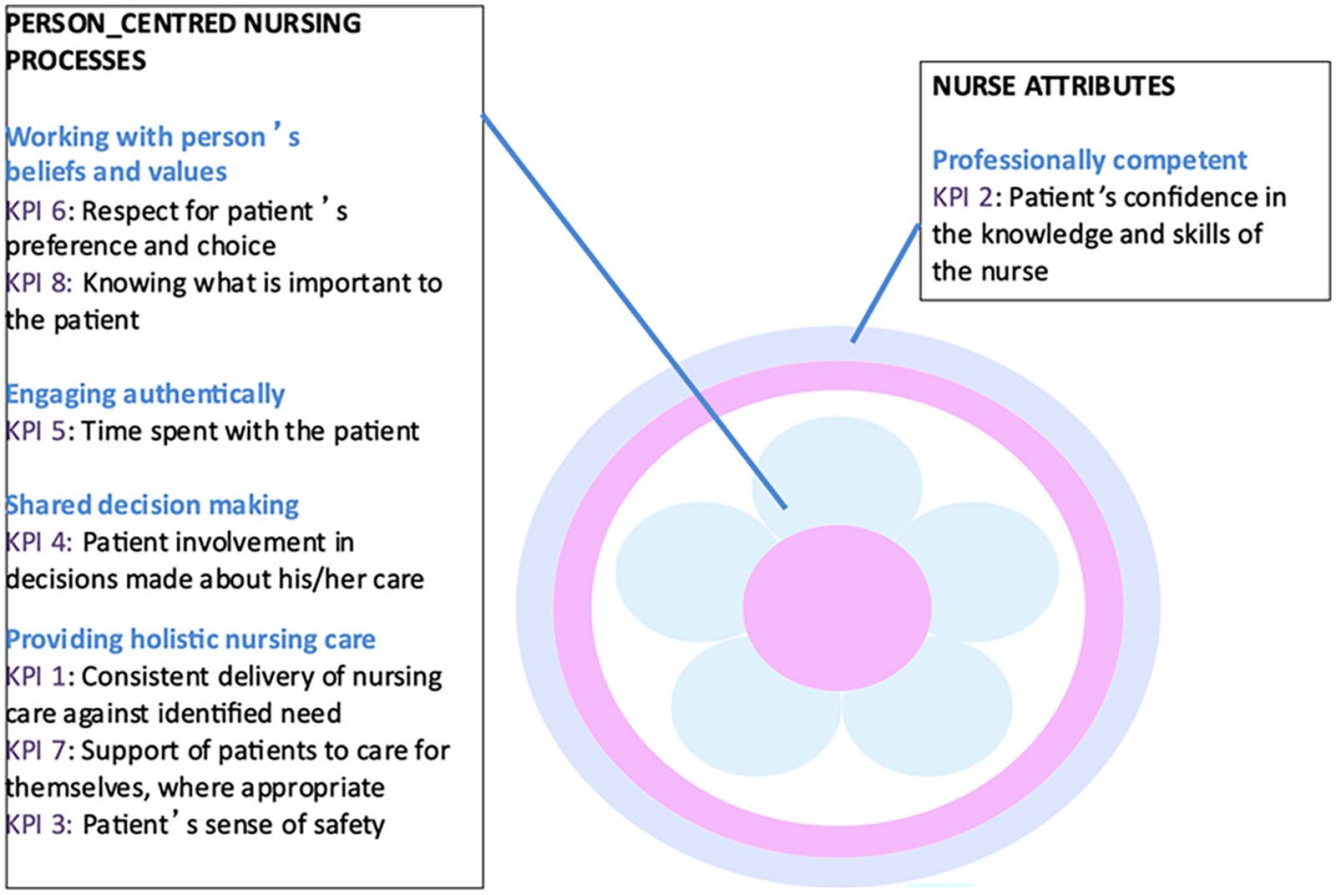
Nursing KPIs mapped to the Person-centred Nursing Framework.

The distinctness of the person-centred processes to the concept of nursing can be further evidenced through a related research programme underpinned by the PCNF focusing on the development and implementation of a set of eight nursing and midwifery key performance indicators (McCance et al., 2012). The indicators, identified from original research, were considered novel in that they: (i) did not conform to most other nursing metrics generally reported in the international literature; (ii) were strategically aligned to work on improving patient experience and (iii) measured person-centred practice. The KPIs are strongly aligned to the person-centred nursing processes, as illustrated in Figure 2, and at the heart of the KPIs is the unique contribution of nursing and ultimately its impact on patient outcomes.

The measurement tools developed to accompany the KPIs comprise: a patient survey; a tool to observe time spent with patients; patient and family stories and a review of the patient record undertaken in conjunction with nurse interviews (McCance et al., 2015). The eight KPIs and measurement tools have subsequently been tested in a series of international implementation research studies across multiple contexts. The evaluation outcomes to date illustrate that the KPIs are: an effective measure to evidence performance of nursing teams; a powerful driver for improvements in practice and a mechanism that can promote person-centred cultures. The programme of work has increased its impact and reach through the development of the iMPAKT APP that supports nursing teams to collect patient experience data that evidence the unique contribution of nursing, whilst also enabling meaningful practice change that promotes person-centred practice (Lynch and McCance, 2022; McCance et al., 2020a; McCance et al., 2020b; Wilson et al., 2021).

### Concept of environment

Within the framework *creating a healthful culture* reflects the extent to which the care environment supports and maintains person-centred principles and is described as one in which decision-making is shared, staff relationships are collaborative, leadership is transformational and innovative practices are supported. McCormack et al. (2011) suggested that contextual factors, such as organisational culture, the learning environment and the care environment itself, pose the greatest challenge to person-centredness and the development of cultures that can sustain person-centred care. Further research by Brown and McCormack (2011) brought into stark focus the impact of the environment of care on evidence-informed person-centred practice. Brown and McCormack (2011) studied post-operative pain management practices with older people following abdominal surgery. They found that barriers to effective post-operative pain management were not dependent upon which decision-making tools were used (such as algorithms and protocols), but more to do with the ‘psychological safety’ of the care environment. A psychologically safe care environment is one in which nurses feel safe to give and receive feedback about their practice, where leadership facilitates open and honest dialogue and where the culture supports reflection on practice. This kind of practice culture is consistent with person-centred values and we suggest is critical to the practice of person-centred nursing.

### Concept of health

Within the PCNF we draw on a broader notion of health that reflects living a positive life, which embraces all dimensions of our being, which is reflected in the outcome – a good care experience. This is set within the context of a social model of health that acknowledges the complexities of human experience and the inter-relationships between mind, body and society (Yuill et al., 2010). In understanding a good experience of care, we have focused on the work of Seedhouse (1986) who refers to a set of conditions that enables a person to strive to reach their potential and describes health in relation to ‘foundations for achievement’. The foundations which make up health, according to Seedhouse include: the basic needs of food, drink, shelter, warmth, etc.; access to the widest possible information and the skills and confidence to assimilate this information; and the recognition that an individual is never totally isolated from other people and the external environment, and cannot be fully understood separated from the influence of their environment. This conceptualisation is also supported by work undertaken by Titchen and McCormack on enabling human flourishing (McCormack and Titchen, 2014; Titchen and McCormack, 2010). These authors argue that human flourishing is the overall outcome arising from working in a person-centred way. They argue that when practitioners integrate the creative energies of different forms of knowledge and intelligences, growthful experiences for all (e.g., staff, service users and families) are enabled. Furthermore, when care is experienced in a way that privileges the personhood of all persons, this ultimately leads to positive outcomes for both those receiving care and those delivering care.

The experience of good care reflects the evaluation that a patient, or indeed a nurse, places on her or his care experience, resulting from nurses with attributes that enable them to manage the care environment to provide person-centred care. We need to emphasise the importance of this outcome being evaluated from the perspective of either patients or nurses or both and indeed the complexity associated with this endeavour. There has been significant development of a range of tools that align to the PCNF to enable the evaluation of the relationship between a person-centred approach to nursing and the resulting outcomes for patients and nurses. Examples include the Person-centred Practice Inventories for Staff (Slater et al., 2017), students (O’Donnell et al., 2021) and patients (McCormack et al., 2024), the Workplace Critical Culture Analysis Tool-revised (Wilson et al., 2020) and the nursing and midwifery KPIs previously discussed. There is, however, still much to be achieved in outcome evaluation, recognising the need to be clear about concepts in order to embrace theory-driven evaluation that often requires multiple data sources as opposed to one definitive measure (McCormack, 2022).

## The impact

Taking cognisance of the complexity associated with the evaluation of person-centred nursing and evidencing outcomes, pathways to impact have been integral to the ongoing development of the PCNF and PCPF. Impact can be evidenced across the areas of policy, strategy, education and practice at a global level, and this has been tracked through the submission of three impact case studies in the last two assessments of research quality in the United Kingdom – Research Assessment Exercise in 2016 and Research Excellence Framework in 2021. The detail of these impacts can be found in the REF results website https://results2021.ref.ac.uk/profiles/units-of-assessment/3. The impacts shown in these case studies can be summarised as demonstrating sustained changes in a wide variety of practice contexts, bringing about outcomes for patients, families and nurses, illustrated by systematic evaluations derived from multiple methodological perspectives and using mixed methods and participatory research designs.

However, for the purpose of this paper, it is useful to provide examples from a series of implementation studies that have focused on the development of person-centred nursing in a variety of practice settings. The PCNF has been used to structure these implementation studies and through using the framework in this way, relationships between concepts have been identified and refined and led to the development of new areas of research. However, it is important to emphasise that the claims made regarding outcomes are study specific and are embedded in different methodological approaches, such as practice development and action research. Nonetheless, the collective body of evidence comprising multiple studies over more than 20 years provides evidence of impact that is significant for future work at systems level.

Implementation studies have been undertaken in aged care and have evidenced improvements to the care environment, greater resident satisfaction, improved staff well-being, reduction in falls and reduced use of psychotropic medications (Buckley et al., 2014; McCormack et al., 2010; Mekki et al., 2017). Studies undertaken in acute care have evidenced better engagement between staff and patients as well as improved retention of staff, greater job satisfaction and staff well-being (Hahtela et al., 2015; Laird et al., 2015; McCance et al., 2013; Parlour et al., 2014). In the area of palliative care, studies have evidenced improvements in regulator quality indicators, as well as improvements to the quality of the care environment, better and more effective staff communication, increased staff development and better retention of staff (Haraldsdottir et al., 2020; McCormack et al., 2018; Yalden et al., 2013). In these studies, the framework has been used to promote an increased understanding of person-centred nursing, with the aim of enabling practitioners to recognise key elements in their practice, generate meaning from data that can inform the development of person-centred nursing and most importantly to focus the implementation and evaluation of developments in practice.

Additionally, several intervention studies have been undertaken, which have reinforced the impact of ‘care environment’/context on implementation and highlighted the essential facilitation strategies required when developing person-centredness in nursing practice. These studies further emphasise the need for embedded realist evaluations and intervention adaptation (Harvey et al., 2018; Macgregor et al., 2023; Morris et al., 2023; Sampson et al., 2020; Seers et al., 2018). The development of large-scale intervention studies is a key focus of future research to further strengthen the evidence base for developing person-centred nursing that leads to a good care experience for everyone.

## Conclusion

This paper presents the PCNF, a model that has been developed from practice, for use in practice. The framework highlights the complexity of person-centred nursing, and through the articulation of the key constructs, emphasises the contextual, attitudinal and moral dimensions of humanistic caring practices. The relationship between the constructs describes the necessity for competent nurses, who have the ability to manage the numerous contextual and attitudinal factors that exist in care environments, to engage in processes that keep the person at the centre of caring interactions. The PCNF is described as a mid-range theory for nursing practice and is clearly positioned within the hierarchy of nursing knowledge. Finally, drawing on the growing evidence base focusing on implementing person-centred nursing through the framework, there is evidence of key outcomes demonstrating an enhanced care experience for both patients and nurses.

**Key points for policy, practice and/or research**

- Person-centredness has a long association with nursing because it prioritises the human experience and places compassion, dignity and humanistic caring principles at the centre of healthcare delivery.
- Evidence demonstrates that development of person-centredness in practice (as opposed to solely focusing on person-centred care) requires strategies to overcome the cultural and contextual challenges to implementing person-centred nursing.
- The Person-centred Nursing Framework is described as a middle-range theory and can be clearly positioned in the context of the metaparadigm of nursing, thus drawing out its uniqueness to the discipline of nursing.
- Evidence of outcomes arising from the implementation of person-centred nursing through the framework, demonstrate an enhanced care experience for both patients and nurses.
